# 2D Intraoperative Ultrasound in Brain Metastasis Resection: A Matched Cohort Analysis from a Single-Center Experience

**DOI:** 10.3390/cancers17142272

**Published:** 2025-07-08

**Authors:** Octavian Mihai Sirbu, Alin Chirtes, Mircea Radu Gorgan, Marian Mitrica

**Affiliations:** 1 Clinical Neuroscience Department, Faculty of Medicine, “Carol Davila” University of Medicine and Pharmacy, Dionisie Lupu Street, No. 37, Sector 2, 020021 Bucharest, Romaniamarian.mitrica@umfcd.ro (M.M.); 2 Doctoral School, Faculty of Medicine, “Carol Davila” University of Medicine and Pharmacy, 050474 Bucharest, Romania; 3 Department of Neurosurgery, ‘Dr. Carol Davila’ Central Military Emergency University Hospital, 010825 Bucharest, Romania; 4 Department of Neurosurgery, “Bagdasar-Arseni” Clinical Emergency Hospital, Berceni Street, No. 12, Sector 4, 041915 Bucharest, Romania

**Keywords:** brain metastases, intraoperative ultrasound, extent of resection, gross total resection, real-time imaging

## Abstract

**Background**: Intraoperative ultrasound (IOUS) provides real-time imaging during brain tumor surgery but remains underused in brain metastasis resection. This study evaluates the effectiveness of 2D IOUS in improving the extent of resection compared to standard neuronavigation. **Methods**: We retrospectively analyzed 55 adult patients with brain metastases treated surgically at a single center. Patients were divided into two groups: IOUS-guided surgery (n = 20) and standard neuronavigation (n = 35). Gross total resection (GTR) was defined as the extent of resection > 96%, assessed volumetrically. Statistical analyses included chi-square tests, logistic regression, and ROC curve analysis. **Results**: GTR > 96% was achieved in 80% of IOUS-guided cases compared to 42.86% in the control group (*p* = 0.008). IOUS significantly increased the odds of achieving GTR (OR = 5.33, *p* = 0.011). Larger tumor volume reduced the likelihood of GTR (OR = 0.469, *p* = 0.025), but this effect was mitigated by IOUS use (interaction OR = 1.986, *p* = 0.044). The regression model showed excellent discrimination (AUC = 0.930, *p* < 0.001). Functional outcomes improved postoperatively in both groups. **Conclusions**: 2D IOUS significantly enhances the extent of resection in brain metastasis surgery, including that for larger tumors. Its accessibility, real-time feedback, and low cost support its wider adoption in neurosurgical practice, especially in settings with limited resources.

## Simple Summary

Brain metastases are a common and serious complication in patients with cancer. Surgery can help relieve symptoms and improve quality of life, especially when the tumor is completely removed. However, standard surgical tools sometimes struggle to show the full extent of the tumor during the operation. This study explores the use of real-time ultrasound imaging during surgery as an additional tool to guide tumor removal. We compared patients who had surgery with ultrasound guidance to those who had conventional surgery using standard navigation tools. Our results showed that ultrasound helped surgeons remove more of the tumor, even in larger cases, without increasing complications. These findings suggest that ultrasound, a low-cost and accessible technique, could help improve surgical outcomes for patients with brain metastases, especially in hospitals with limited resources. This study may encourage wider use of intraoperative ultrasound in brain tumor surgery and support further research on its benefits.

## 1. Introduction

Brain metastases represent the most common intracranial tumors in adults, occurring in up to 30% of patients with systemic cancer and significantly affecting quality of life and survival [1]. Surgical resection remains a cornerstone of treatment, particularly in cases of solitary or symptomatic lesions, with the goal of achieving gross total resection (GTR) while minimizing neurological morbidity [2].

While image-guided neuronavigation has become a standard adjunct in modern neurosurgery, its accuracy can be compromised by brain shift during dura opening, cerebrospinal fluid (CSF) draining, or ongoing resection [3,4]. Intraoperative ultrasound (IOUS) offers real-time imaging and is a cost-effective, portable modality that allows dynamic assessment of residual tumor during surgery. Despite its potential, IOUS remains underutilized in many centers, and its comparative value relative to standard neuronavigation continues to be debated [5,6,7].

The application of IOUS has been extensively studied in glioma surgery, where it has proven effective in enhancing the extent of resection (EOR) and identifying residual tumor tissue [8,9,10]. However, its role in the surgical management of cerebral metastases remains far less explored [11,12,13]. Most existing data derive from earlier studies conducted in the 1990s and early 2000s, which predate significant advances in ultrasound resolution, surgical technique, and perioperative management [14,15,16]. Given the evolving imaging technology and growing emphasis on individualized oncologic care, extrapolating findings from glioma surgery to metastatic tumors may not be appropriate, and updated data specific to brain metastases are critically needed.

This retrospective study evaluates the efficacy of 2D IOUS in guiding GTR of brain metastases, comparing outcomes with a matched cohort operated using conventional neuronavigation alone. By assessing resection rates, EOR, and postoperative outcomes, we aim to highlight the practical benefits and limitations of IOUS as a standalone or complementary tool in the resection of cerebral metastases.

## 2. Materials and Methods

This retrospective observational study analyzed 55 patients who underwent surgical resection for brain metastases at a single neurosurgical center.

Patients included in this study were adults (≥18 years) with histologically confirmed brain metastases who underwent surgical resection with GTR intent. Eligibility required the availability of both preoperative and early postoperative imaging suitable for volumetric assessment, as well as complete intraoperative and postoperative clinical data. All included patients had a solitary or dominant symptomatic lesion and a preoperative Karnofsky Performance Status (KPS) of 60 or higher.

Patients were excluded if they had diffuse leptomeningeal dissemination, underwent reoperation for previously treated lesions, or had incomplete imaging data that precluded accurate tumor volume analysis. Additional exclusion criteria included emergency procedures performed without oncologic resection intent (e.g., for hemorrhagic decompression), cases treated by biopsy alone or subtotal debulking without resection attempt, the presence of severe systemic illness impairing postoperative evaluation, and cases lost to immediate postoperative follow-up.

Demographic and clinical variables were collected, including sex, age, KPS, primary tumor type, lesion location and laterality, pre- and postoperative tumor volumes, and hospital stay duration. The presence of neurological deficits and the onset time of symptomatology were also recorded.

Patients were divided into two cohorts based on whether IOUS was used during surgery: the IOUS group (n = 20) and the non-IOUS group (n = 35). The choice of using either method was taken by the treating surgeon based on technique familiarity or logistical constraints.

Postoperative evaluation of the EOR was performed using contrast-enhanced cranial CT on postoperative day 1, at the time of patient transfer from the intensive care unit to the neurosurgical ward. Tumoral volume assessment was made by volumetric semi-automated segmentation using free open-source software 3D-Slicer [17].

Statistical analysis was performed using IBM SPSS Statistics version 27 and Microsoft Excel 2019. The normality of distributions was assessed with the Kolmogorov–Smirnov and Shapiro–Wilk tests. Depending on variable type and distribution, Mann–Whitney U tests, Wilcoxon signed-rank tests, Chi-square tests, Fisher’s exact test, and binary logistic regression were used. ROC analysis was used to evaluate model discrimination. Statistical significance was set at *p* < 0.05.

## 3. Results

### 3.1. Demographics and Baseline Characteristics (Table 1)

Most patients were male (60%), with a mean age of 61.82 years (SD: 10.03; CI: [59.11; 64.53]). No significant differences were found between the IOUS and non-IOUS groups in terms of age [IOUS group: 62.40 years old (SD: 9.45; CI: [57.98; 66.82]) Non-IOUS group: 61.49 ani (SD: 10.46; CI: [57.89; 65.08]); *p* = 0.742], sex distribution (*p* = 0.567), or preoperative KPS [IOUS group: 76.50 (SD: 11.37; CI: [71.18; 81.82]); Non-IOUS group: 76.29 (SD: 11.14; CI: [72.46; 80.11]); *p* = 0.833]. Most common symptoms included headache (54.55%) and motor deficits (41.82%). Symptom onset prior to surgery was shorter in the IOUS group (mean 4.06 vs. 7.29 weeks, *p* = 0.117).

**Table 1:** Patients’ characteristics.

Preoperative Lot Characteristics	Total	IOUS Group	Non-IOUS Group
No. of patients	55	20	35
Mean age (years old)	61.82	62.40	61.49
Sex distribution (Male)	60%	65%	57%
Duration of symptoms (mean weeks)	6.11	4.06	7.28
Acute presentation with intracranial hypertension syndrome	18.18%	25.00%	14.29%
Symptomatology ∘ Headache ∘ Motor deficits ∘ Dizziness ∘ Epilepsy	54.55% 41.82% 41.82% 20.00%	55.00% 50.00% 35.00% 20.00%	54.29% 37.14% 45.71% 20.00%
Lesion location ∘ Frontal lobe ∘ Parietal lobe ∘ Temporal lobe ∘ Occipital lobe ∘ Posterior fossa	26.14% 24.95% 14.26% 11.88% 17.81%	33.93% 20.36% 13.57% 6.79% 20.36%	21.92% 27.39% 14.62% 14.62% 16.45%
Lateralization (left side)	29.09%	25.00%	31.43%
Primary tumor type • Lung cancer (NSCLC) • Skin melanoma • Prostate adenocarcinoma • Breast Carcinoma • Colorectal adenocarcinoma • Unknown	45.45% (31.42%) 5.45% 3.64% 10.91% 5.45% 25.45%	55% (45%) 10% 10% - - 25%	40% (36.36%) - - 17% 9% 25.71%

### 3.2. Tumor Characteristics

Lung cancer was the most frequent primary tumor (45.45%), particularly in the IOUS group (55%), with Non-Small Cell Lung Carcinoma (NSCLC) being the most frequent subtype. Preoperative tumor volume was higher in the IOUS group (mean 17.6 cm^3^ vs. 11.7 cm^3^), though the difference was not statistically significant (*p* = 0.234). Lesion locations were evenly distributed between groups, with the frontal and parietal lobes being most affected.

### 3.3. Extent of Resection and GTR

**Figure 1:**
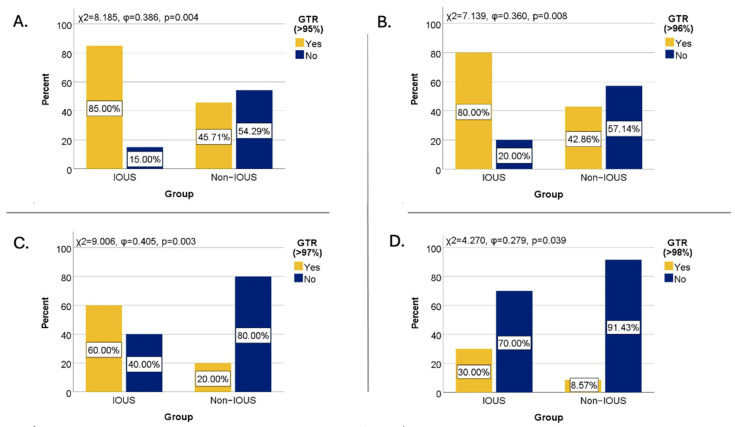
GTR rate based on IOUS use at different GTR cutoffs: (**A**)—95%; (**B**)—96%; (**C**)—97%; (**D**)—98%.

Using a 95% threshold, GTR was achieved in 85% of IOUS-guided surgeries, in contrast to only 45.71% of surgeries performed without IOUS. Chi-square and Fisher’s tests confirmed a statistically significant, direct, and moderately strong association between IOUS use and achieving GTR > 95% (χ^2^ = 8.185, φ = 0.386, *p* = 0.004; Fisher *p* = 0.005). The odds of achieving complete resection were approximately seven times higher when IOUS was used (OR = 6.73, 95% CI: [1.67–27.18], *p* = 0.007) (Figure 1A).

Using a 96% EOR cutoff, GTR was achieved in 80% of cases operated with IOUS (n = 16, 4 out of 5 cases), compared to 42.86% of those operated without IOUS (n = 15, 2 out of 5 cases). Chi-square and Fisher’s exact tests revealed a statistically significant and moderately strong association between IOUS use and achieving GTR > 96% (χ^2^ = 7.139, φ = 0.360, *p* = 0.008). The odds of achieving GTR > 96% were approximately five times higher when IOUS was used (OR = 5.33, *p* = 0.011) (Figure 1B).

At a 97% threshold, GTR was achieved in 60% of patients in the IOUS group, while only 20% of patients in the non-IOUS group reached this extent of resection. Chi-square and Fisher’s exact tests revealed a statistically significant and moderately strong association between IOUS use and GTR > 97% (χ^2^ = 9.006, φ = 0.405, *p* = 0.003). The odds of achieving this resection threshold were six times higher in surgeries performed with IOUS (OR = 6.00, *p* = 0.004) (Figure 1C).

Using a 98% EOR cutoff, GTR was achieved in 30% of cases operated with IOUS, compared to only 8.57% of cases operated without IOUS. The Chi-square test indicated a statistically significant but weak association between IOUS use and the achievement of GTR > 98% (χ^2^ = 4.270, φ = 0.279, *p* = 0.039). However, the odds ratio for this threshold was not statistically significant (*p* = 0.055), suggesting limited predictive strength at higher EOR cutoffs (Figure 1D).

A binary logistic regression analysis was performed to assess the influence of IOUS use, preoperative tumor volume, and their interaction (IOUS × preoperative tumor volume) on the likelihood of achieving an extensive tumor resection (GTR > 96%).

**Figure 2:**
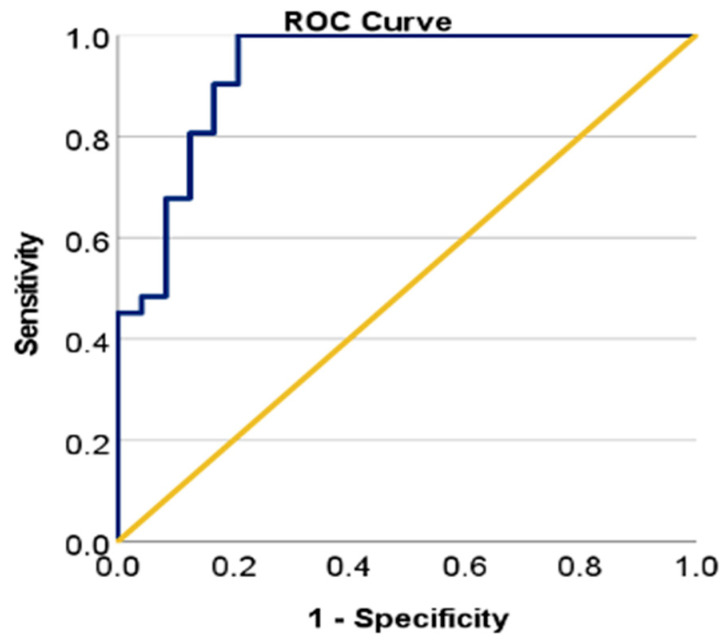
The ROC curve indicated excellent performance of the previously proposed regression model in discriminating cases with GTR > 96%, with an AUC of 0.930 (CI: [0.860; 1], *p* < 0.001).

The logistic regression model was statistically significant (χ^2^ = 46.353, *p* < 0.001), demonstrating strong predictive power. The model explained between 54.5% (Cox & Snell) and 73.1% (Nagelkerke) of the variance in the probability of achieving GTR > 96%. The Hosmer & Lemeshow goodness-of-fit test indicated a good model fit to the data (*p* = 0.506). The model achieved a correct classification rate of 89.1%, indicating very good to excellent accuracy (Figure 2).

Preoperative tumor volume was negatively associated with achieving GTR > 96%, with larger tumor volumes decreasing the likelihood of complete resection (OR = 0.469, 95% CI: [0.242–0.910], *p* = 0.025).

The interaction term between tumor volume and IOUS use indicated that IOUS mitigates the negative effect of larger tumor size on the likelihood of achieving GTR, suggesting that IOUS increases the probability of GTR even in larger metastases (OR = 1.986, 95% CI: [1.019–3.872], *p* = 0.044).

### 3.4. Functional Outcomes (Table 2)

Preoperative KPS was similar in both groups, with a mean of 76.36 (SD: 11.12; CI: [73.36; 79.37]). KPS scores improved significantly postoperatively in both groups (IOUS group: 82.00 (SD: 8.34; CI: [77.30; 86.70]); Z = 3.051, *p* = 0.002; non-IOUS: 77.71 (SD: 10.03; CI: [74.34; 81.08]) Z = 2.236, *p* = 0.025). No significant difference was found in postoperative KPS between groups (*p* = 0.182). Postoperative discharge time was similar between groups (*p* = 0.130).

**Table 2:** Surgery-related outcomes.

	Total		IOUS Group		Non-IOUS Group	
	PreOp	PostOp	PreOp	PostOp	PreOp	PostOp
KPS	76.36	79.27	76.50	82.00	76.29	77.71
Volume (cc)	13.82	1.13	17.61	0.90	11.66	1.26
EOR (%)	93.70%		96.30%		92.21%	
GTR (>95%)	60%		85%		45.71%	
Postoperative stay (days)	4.61		5.25		4.24	

## 4. Discussion

This study reinforces the utility of 2D IOUS as an accessible and effective tool in the surgical management of brain metastases. While extensively validated in glioma surgery, its role in metastatic lesions remains less defined [8,9,10,17]. Our findings suggest that IOUS significantly enhances GTR rates, including in patients with larger tumor volumes, underscoring its practical clinical impact.

The demographic characteristics of our cohort, including a mean age of 61.8 years and male predominance (60%), align with epidemiologic trends reported in population-level analyses such as SEER and studies by Nayak et al. [1,18]. Similarly, the mean preoperative Karnofsky Performance Status (KPS) was 76.4, consistent with commonly accepted surgical inclusion thresholds [17]. These aspects reinforce the external validity of our findings and the generalizability of our conclusions.

In terms of baseline functional status, the preoperative Karnofsky Performance Score (KPS) averaged 76.4 across the cohort, with no significant difference between the IOUS and non-IOUS groups (*p* = 0.833). This aligns with the inclusion criteria commonly reported in neurosurgical series, which typically require a minimum KPS of 60–70 for surgical candidacy [19]. Overall, our cohort appears demographically consistent with previously published data, reinforcing the external validity of our findings. The balanced distribution of key demographic variables between groups also supports the robustness of the comparative analysis between IOUS and non-IOUS interventions.

Multiple intraoperative tools have been developed to improve the safety and extent of brain tumor resection, including intraoperative MRI (iMRI), intraoperative CT (iCT), fluorescein sodium (FS), 5-aminolevulinic acid (5-ALA), and IOUS. iMRI offers high-resolution imaging and real-time anatomical updates but is associated with significant logistical demands, prolonged operative time, and high infrastructure costs [20,21]. 5-ALA enables selective fluorescence of high-grade gliomas under blue light, enhancing visualization of tumor margins, though it is limited by regulatory access and cost. FS is a more affordable and broadly available alternative, providing useful contrast particularly in metastases and gliomas with disrupted blood–brain barrier [22]. Several studies demonstrated that sodium fluorescein-guided resection of brain metastases from lung cancer significantly improves EOR (87.0% vs. 62.1%) and reduces local recurrence (8.7% vs. 34.5%) [22]. Similarly, our study supports the use of IOUS as an effective real-time tool to enhance tumor delineation and optimize resection. Both techniques serve the shared goal of maximizing resection while preserving neurological function, each with distinct advantages depending on surgical context and resource availability.

FS and IOUS, while operator-dependent, remain some of the most cost-effective and flexible modalities, allowing real-time assessment of tumor extent and residual tissue without the need for major equipment [23,24]. Moreover, their combined use and complementary have also been reported. A systematic review by Di Cristofori et al. analyzed the combined use of IOUS and fluorescence-guided surgery in brain metastases [11]. The authors emphasized the lack of large, focused studies on IOUS as a standalone modality in metastasis resection, highlighting the need for clearer reporting of EOR and GTR outcomes specific to tumor histology.

While IOUS is well-established in glioma surgery, its application in the resection of brain metastases remains underexplored in the literature. Most existing publications on IOUS either focus exclusively on high-grade gliomas or include mixed cohorts of intracranial tumors, with subgroup analyses for brain metastases often absent or insufficiently detailed [17].

A systematic review by Di Cristofori et al. analyzed the combined use of IOUS and fluorescence-guided surgery in brain metastases [11]. The authors emphasized the lack of large, focused studies on IOUS as a standalone modality in metastasis resection, highlighting the need for clearer reporting of EOR and GTR outcomes specific to tumor histology. Our cohort, consisting of 55 patients (20 with IOUS-guided surgery), contributes to addressing this gap by offering focused, volumetric, and comparative data on surgical outcomes in brain metastasis cases.

In this context, our study—with 55 patients, including 20 in the IOUS group—represents one of the largest single-center, metastasis-focused cohorts comparing IOUS-guided surgery with standard neuronavigation [12,16]. Unlike broader mixed studies, our methodology allowed for direct comparisons of EOR, GTR, KPS, and postoperative recovery specifically in the setting of metastatic brain disease. The relatively large sample size, robust statistical design, and use of multiple GTR thresholds strengthen the generalizability of our results and address an unmet need in the current literature.

Moreover, the rarity of exclusive IOUS studies in brain metastases is likely multifactorial, owing to the traditional emphasis on palliation over radical resection in metastatic disease and the heterogeneity of primary tumors. However, recent shifts in neuro-oncological philosophy emphasize maximal safe resection even in metastases, especially for patients with controlled systemic disease and favorable functional status (KPS ≥ 70), further underscoring the importance of imaging adjuncts such as IOUS.

In our study, IOUS was employed in its most accessible form: conventional 2D B-mode imaging, without contrast enhancement, 3D volumetric reconstruction, or integration with navigational systems. Despite these limitations, our results demonstrated significant improvements in EOR and GTR rates in the IOUS group compared to controls.

Recent advances in IOUS technology offer several enhancements beyond 2D imaging. Navigated IOUS (nIOUS) integrates real-time ultrasound data into neuronavigation platforms, effectively compensating for brain shift and improving anatomical accuracy. Several studies have shown that nIOUS can refine tumor margin delineation and correlate favorably with intraoperative MRI in glioma surgery [17,25,26,27]. However, such systems require advanced hardware, calibration procedures, and substantial institutional investment.

3D IOUS enables volumetric tumor assessment and multiplanar resection control. While promising, its utility in metastasis surgery remains underreported. Some authors have described the benefits of 3D ultrasound in guiding resection in both gliomas and metastases, though image acquisition and reconstruction times may limit its intraoperative flexibility [25,28].

Contrast-enhanced IOUS (CEUS) introduces ultrasound contrast agents (microbubbles) to better visualize tumor vascularity and residual enhancing tissue. Preliminary reports suggest that CEUS may improve tumor margin definition, especially in hypervascular metastases such as melanoma or renal cell carcinoma. However, CEUS remains off-label for brain applications in many countries and lacks standardized protocols [7,29,30].

The use of 2D B-mode IOUS alone, as in our study, offers a low-cost, portable, and real-time solution, particularly suited for resource-limited settings. Our findings reinforce that even without advanced adjuncts, IOUS remains a valuable tool in enhancing resection quality. Nevertheless, further prospective studies are needed to delineate the added value of navigated, 3D, and contrast-enhanced IOUS specifically in the context of brain metastasis surgery.

### Limitations

This study has several limitations. Its retrospective, non-randomized design limits causal inference, although baseline comparability between groups was ensured. The sample size, particularly in the IOUS group (n = 20), may appear limited, yet it remains comparable to other published series focused exclusively on brain metastases and IOUS guidance [11,12]. Prospective designs in this area remain rare due to ethical and logistical constraints, especially given the heterogeneity of systemic oncologic treatments and variable surgical indications.

One limitation of our study is the use of postoperative contrast-enhanced CT instead of MRI; however, in a resource-limited setting, this approach—combined with the use of IOUS—represents a pragmatic and cost-effective strategy to assess and optimize the EOR within existing infrastructure.

In addition, volumetric assessment was performed using 3D Slicer software, which, despite being validated and widely used in neurosurgical research, remains subject to operator-dependent variability, particularly in delineating tumor margins on postoperative imaging. This introduces a degree of subjectivity that may influence the calculated EOR, especially in cases with minimal residual enhancement or postoperative changes.

We also acknowledge that only 2D B-mode IOUS was used, without navigational integration, contrast enhancement, or 3D reconstruction. While this reflects a realistic scenario in many neurosurgical settings, it may underestimate the full potential of more advanced ultrasound technologies. Lastly, the absence of long-term follow-up data limits assessment of recurrence or survival impact, which remain important endpoints in the broader management of brain metastases.

## 5. Conclusions

Our findings support the role of 2D intraoperative ultrasound as an effective and accessible adjunct in the resection of brain metastases. Its use was associated with significantly higher rates of gross total resection, particularly in larger tumors, without negatively impacting functional outcomes. Even in the absence of advanced technologies such as navigation, contrast enhancement, or 3D reconstruction, conventional 2D IOUS provides meaningful intraoperative guidance that can improve surgical precision. Given its low cost, real-time feedback, and portability, IOUS may be especially valuable in centers with limited resources.

## Data Availability

The raw data supporting the conclusions of this article will be made available by the authors on request.

## Abbreviations

The following abbreviations are used in this manuscript:

5-ALA	5 aminolevurinic acid
CEUS	Contrast-enhanced ultrasonography
CSF	Cerebrospinal fluid
EOR	Extent of resection
GTR	Gross total resection
iCT	Intraoperative CT
iMRI	Intraoperative MRI
IOUS	Intraoperative ultrasound
KPS	Karnofsky performance scale
nIOUS	navigated IOUS
NSCLC	Non-small cell lung carcinoma
SF	Sodium fluorescein
